# Nitric Oxide Alleviates Low-Light-Induced Photosynthetic Impairment by Improving Photosystem Coordination and Electron Transport in Tomato Seedlings

**DOI:** 10.3390/plants15172668

**Published:** 2026-08-31

**Authors:** Xianjun Chen, Bi Chen, Lingling Hu, Zhuang Wen, Mingjie Liu, Qin Yang, Huiying Liu

**Affiliations:** 1 Provincial Famous Teacher Yang Qin Studio/Guizhou Key Laboratory of Molecular Breeding for Characteristic Horticultural Crops, School of Life and Health Science, Kaili University, Kaili 556011, China; chenxianjun_0805@163.com (X.C.); 18487194930@163.com (B.C.); 19119078027@163.com (L.H.); gzu_zwen@163.com (Z.W.); liumingjiee@163.com (M.L.); 2 Key Laboratory of Special Fruits and Vegetables Cultivation Physiology and Germplasm Resources Utilization of Xinjiang Production and Contruction Crops, Department of Horticulture, Agricultural College, Shihezi University, Shihezi 832003, China

**Keywords:** tomato seedlings, low light, nitric oxide, photosynthetic electron transport, photosystem coordination, cyclic electron flow

## Abstract

Low light is a major environmental constraint that limits crop productivity by restricting photosynthetic carbon assimilation and disrupting photosynthetic function. Nitric oxide (NO) is an important signaling molecule involved in plant stress responses; however, its role in regulating photosystem stability under low-light conditions remains unclear. In this study, tomato seedlings were treated with the NO donor sodium nitroprusside (SNP) and the NO scavenger 2-phenyl-4,4,5,5-tetramethylimidazoline-1-oxyl-3-oxide (PTIO) to investigate the effects of NO-related treatments on growth, photosynthetic performance, photosystem function, electron transport, ROS accumulation, and antioxidant defense under low-light conditions. Low light markedly suppressed growth and impaired the photochemical activities of both photosystem II (PSII) and photosystem I (PSI), as indicated by decreases in the maximum quantum efficiency of PSII (Fv/Fm), the maximal P700 oxidation capacity (Pm), and electron transport rates, together with enhanced reactive oxygen species (ROS) accumulation. Exogenous NO application significantly improved photosystem performance under low-light conditions. NO enhanced PSII and PSI photochemical performance, improved excitation energy distribution, and restored photosynthetic electron transport, while strengthening antioxidant enzyme activities, thereby reducing excitation pressure and oxidative damage. In contrast, PTIO treatment aggravated photosystem impairment under low light. These findings indicate that NO contributes to maintaining photosystem function and photosynthetic electron transport under low-light conditions, highlighting its potential role in improving low-light tolerance in tomato.

## 1. Introduction

Light is the primary energy source driving photosynthesis and plays an indispensable role in regulating plant growth, organogenesis, and reproductive development [1,2]. However, suboptimal light environments are common in many agricultural production systems, especially in protected cultivation, high-latitude regions, and areas with frequent cloud cover. Prolonged exposure to low-light conditions has become an important environmental constraint limiting plant growth, photosynthetic efficiency, and crop productivity worldwide. Under low-light conditions, carbon assimilation is often more severely restricted than light absorption, resulting in an imbalance between energy utilization and energy dissipation that compromises photosynthetic efficiency and photosystem stability [3,4]. Tomato (*Solanum lycopersicum* L.) is a typical light-demanding horticultural crop whose growth and yield are highly dependent on adequate and stable irradiance. Exposure to low-light stress frequently results in leaf chlorosis, excessive stem elongation, reduced photosynthetic performance, and impaired carbon assimilation [5,6]. Moreover, insufficient light availability restricts the accumulation of photosynthetic assimilates, disrupts floral initiation, and increases the node position of the first inflorescence, thereby adversely affecting flowering, pollination, and fruit set. These physiological and developmental alterations ultimately lead to substantial yield losses in tomato production systems [7]. Therefore, elucidating how tomato maintains photosystem function and photosynthetic performance under low-light conditions is of both fundamental scientific interest and considerable agronomic importance.

Light intensity determines the amount of photosynthetically active radiation available for carbon assimilation and biomass production, thereby setting the upper boundary for plant growth and productivity [8]. Through photosynthesis, plants convert light energy into chemical energy, and the structure and function of the photosynthetic machinery are highly plastic in response to irradiance fluctuations. Under high-light conditions, plants generally increase nitrogen allocation to components involved in photosynthetic electron transport, Calvin–Benson cycle enzymes, and chloroplast ATP synthase to enhance photosynthetic capacity [9,10]. In contrast, low-light acclimation alters the organization and coordination of the photosynthetic apparatus, leading to reduced electron transport capacity and less efficient energy utilization between photosystem II (PSII) and photosystem I (PSI) [11]. Although incident irradiance is limited under low-light conditions, prolonged restriction of carbon assimilation may still disturb the balance between energy absorption and energy consumption, leading to excess excitation energy, reactive oxygen species (ROS) accumulation, and functional impairment of the photosynthetic apparatus [12,13]. To mitigate such damage, plants have evolved sophisticated photoprotective mechanisms, including non-photochemical quenching, alternative electron transport pathways, and antioxidant defense systems, which collectively maintain redox homeostasis and photosystem stability [14,15]. Nevertheless, prolonged low-light exposure may disturb the coordination between light harvesting, electron transport, and carbon assimilation. Such disturbances can impair photosystem function and, under persistent stress, increase the susceptibility of PSI and PSII to photoinhibitory damage once excitation energy can no longer be efficiently utilized or dissipated [16,17].

In addition to intrinsic photoprotective mechanisms, plants rely on various signaling molecules to coordinate physiological and biochemical responses under environmental stress. Nitric oxide (NO) is a small gaseous signaling molecule that has emerged as a key regulator of plant growth, development, and stress adaptation [18,19,20]. Pharmacological approaches using the NO donor sodium nitroprusside (SNP) and the specific NO scavenger 2-phenyl-4,4,5,5-tetramethylimidazoline-1-oxyl-3-oxide (PTIO) have been widely employed to investigate NO functions in plant stress physiology [21,22]. Numerous studies have demonstrated that NO participates in the regulation of photosynthesis, antioxidant metabolism, stomatal behavior, and gene expression under diverse abiotic stresses, including drought, salinity, temperature extremes, and heavy metal toxicity [23,24,25]. Studies have shown that NO also plays an important regulatory role under low-light conditions. For instance, exogenous NO has been reported to increase chlorophyll content and upregulate chlorophyll biosynthesis-related genes in pepper leaves, thereby enhancing photosynthetic performance under low light [26]. Similarly, NO was found to maintain cellular morphology and chloroplast ultrastructure in pakchoi seedlings exposed to low-light stress, ultimately promoting plant growth [27].

However, most previous studies have primarily focused on pigment content, basic photosynthetic parameters, or changes in photosystem II, whereas information regarding electron transport processes and photosystem I activity under low-light conditions remains limited. In particular, whether NO contributes to maintaining PSI and PSII function and alleviating photoinhibitory damage associated with low-light stress remains largely unknown. Therefore, the present study investigated the effects of exogenous NO and PTIO on growth, photosynthetic performance, PSI and PSII function, excitation energy distribution, electron transport, ROS accumulation, and antioxidant defense in tomato seedlings under low-light conditions. By integrating these physiological and biochemical responses, we aimed to clarify the role of NO in maintaining photosynthetic function and redox homeostasis under low-light stress.

## 2. Results

### 2.1. Effects of Low Light and NO on Growth Parameters of Tomato Seedlings

**Figure 1:**
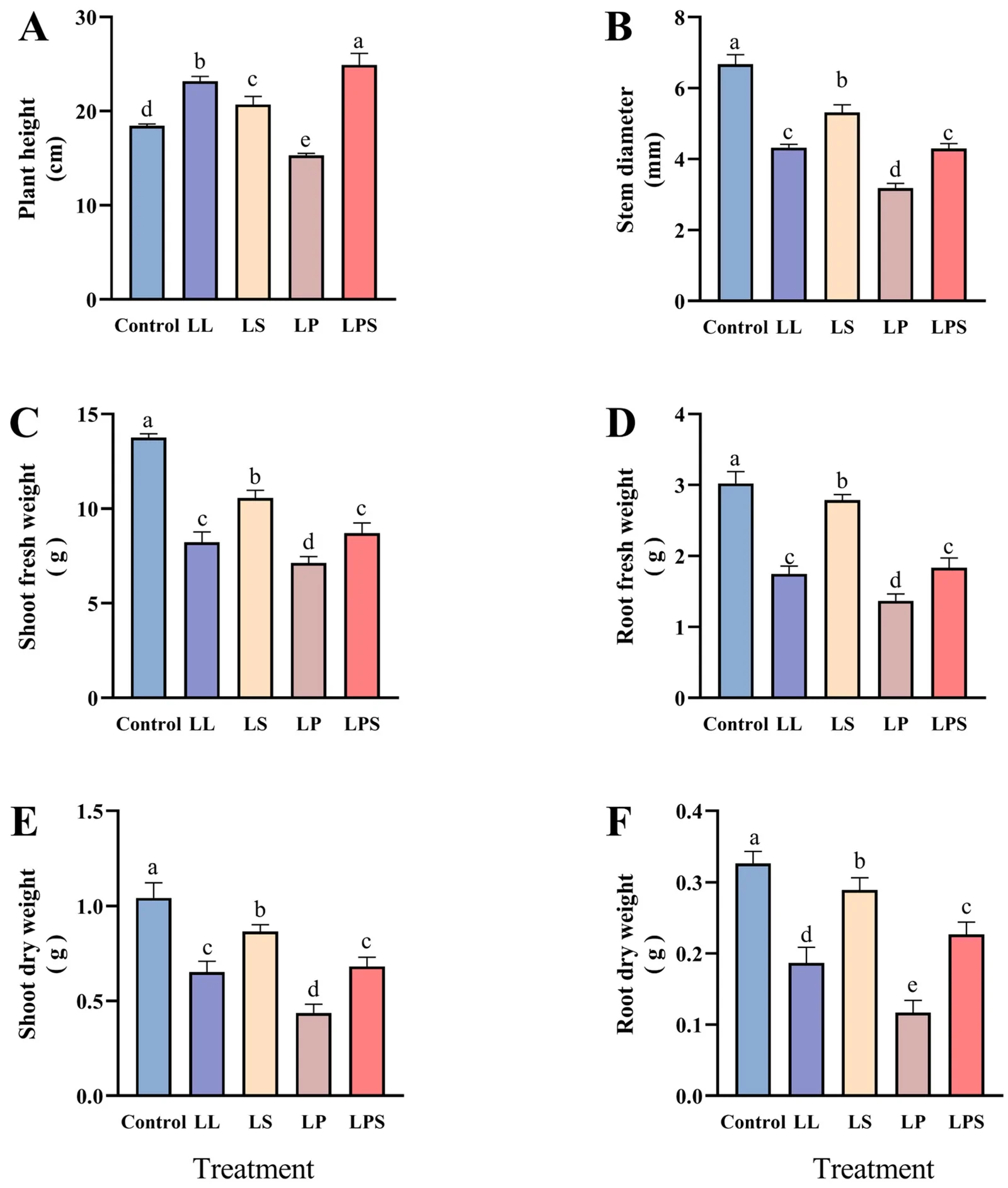
Effects of low light and nitric oxide on tomato seedling growth. Plant height (**A**), stem diameter (**B**), shoot fresh weight (**C**), root fresh weight (**D**), shoot dry weight (**E**), and root dry weight (**F**) were measured under control, low-light (LL), LL + SNP (LS), LL + PTIO (LP), and LL + PTIO + SNP (LPS) treatments. Data are mean ± SD (*n* = 3). Different letters indicate significant differences at *p* < 0.05 (Duncan’s test).

As shown in Figure 1, low-light treatment (LL) significantly increased the plant height of tomato seedlings by 25.7% compared with the control. However, LL markedly reduced stem diameter (35.2%), shoot fresh weight (40.3%), root fresh weight (42.1%), shoot dry weight (37.5%), and root dry weight (42.7%). Exogenous application of sodium nitroprusside (SNP) under low-light conditions significantly alleviated these inhibitory effects, whereas treatment with the NO scavenger PTIO further aggravated the growth suppression induced by LL. Compared with the LP treatment, LPS significantly increased plant height, stem diameter, and both shoot and root fresh and dry weights of tomato seedlings. The contrasting responses of plant height among LL, LP, and LPS may reflect the balance between low-light-induced shoot elongation and the overall inhibition of growth under the combined low-light and PTIO treatment: although low light promoted shoot elongation, the stronger growth inhibition in LP may have constrained this response, whereas SNP treatment in LPS partially alleviated the growth inhibition and allowed the low-light-induced elongation response to become more evident.

### 2.2. Effects of Low Light and NO on Chlorophyll Content in Tomato Seedlings

**Figure 2:**
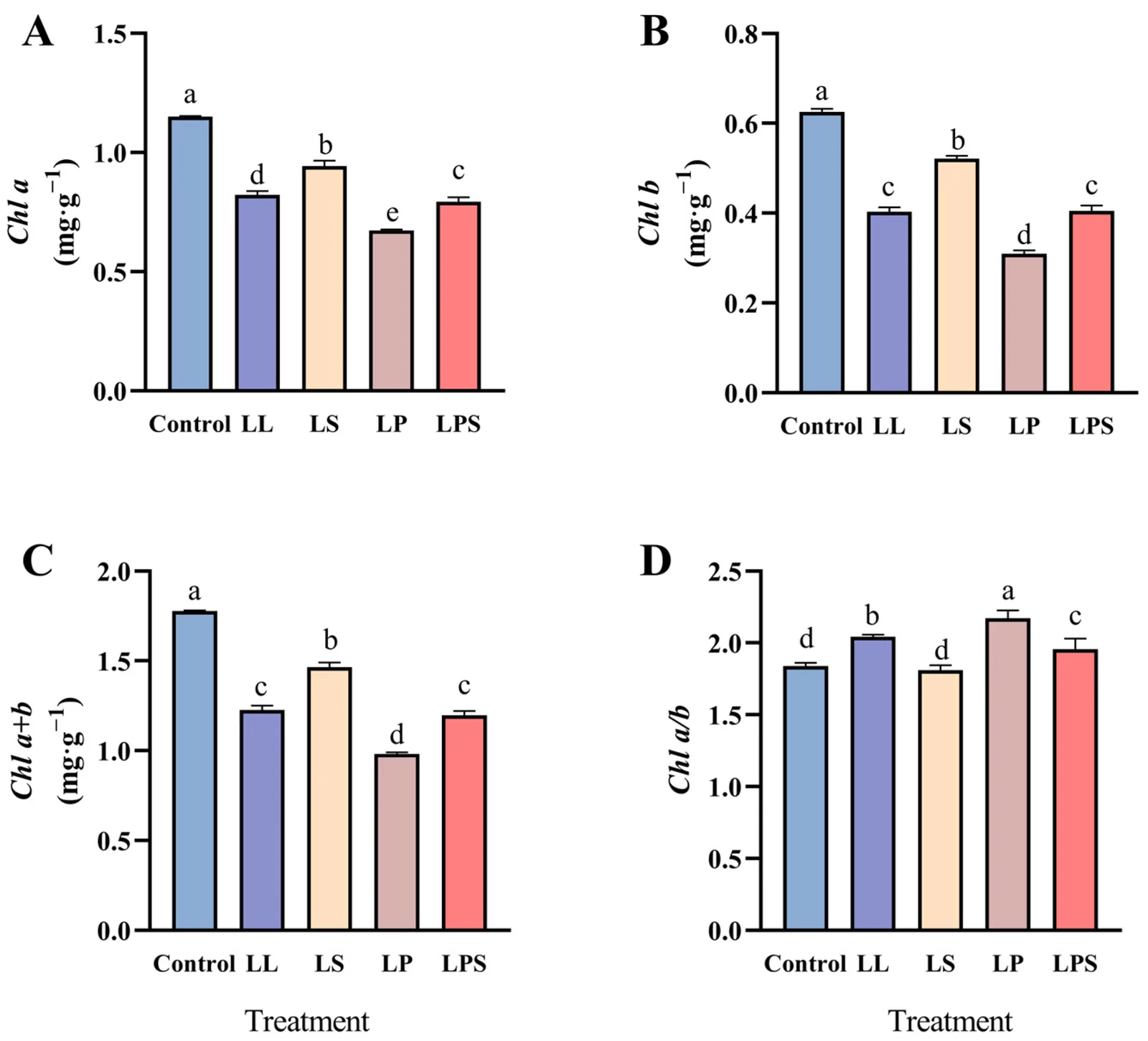
Effects of low light and nitric oxide on chlorophyll content. *Chl a* (**A**), *Chl b* (**B**), *Chl a + b* (**C**), and *Chl a/b* ratio (**D**) were measured under control, low-light (LL), LL + SNP (LS), LL + PTIO (LP), and LL + PTIO + SNP (LPS) treatments. Data are mean ± SD (*n* = 3). Different letters indicate significant differences at *p* < 0.05 (Duncan’s test).

Compared with the control, low-light treatment (LL) significantly reduced chlorophyll a (*Chl a*), chlorophyll b (*Chl b*), and total chlorophyll content (*Chl a *+* b*), while significantly increasing the *Chl a*/*b* ratio (Figure 2). Relative to LL, LS treatment significantly increased *Chl a*, *Chl b*, and *Chl a* + *b* by 14.7%, 29.5%, and 19.6%, respectively, whereas the *Chl a*/*b* ratio decreased by 11.4%. In contrast, LP treatment further reduced *Chl a* (18.2%), *Chl b* (23.1%), and *Chl a* + *b* (19.8%), accompanied by a 6.4% increase in the *Chl a*/*b* ratio. Moreover, compared with LP, LPS treatment significantly increased *Chl a*, *Chl b*, and *Chl a* + *b* contents while decreasing the *Chl a*/*b* ratio.

### 2.3. Effects of Low Light and NO on Photosynthetic Parameters in Tomato Seedlings

**Figure 3:**
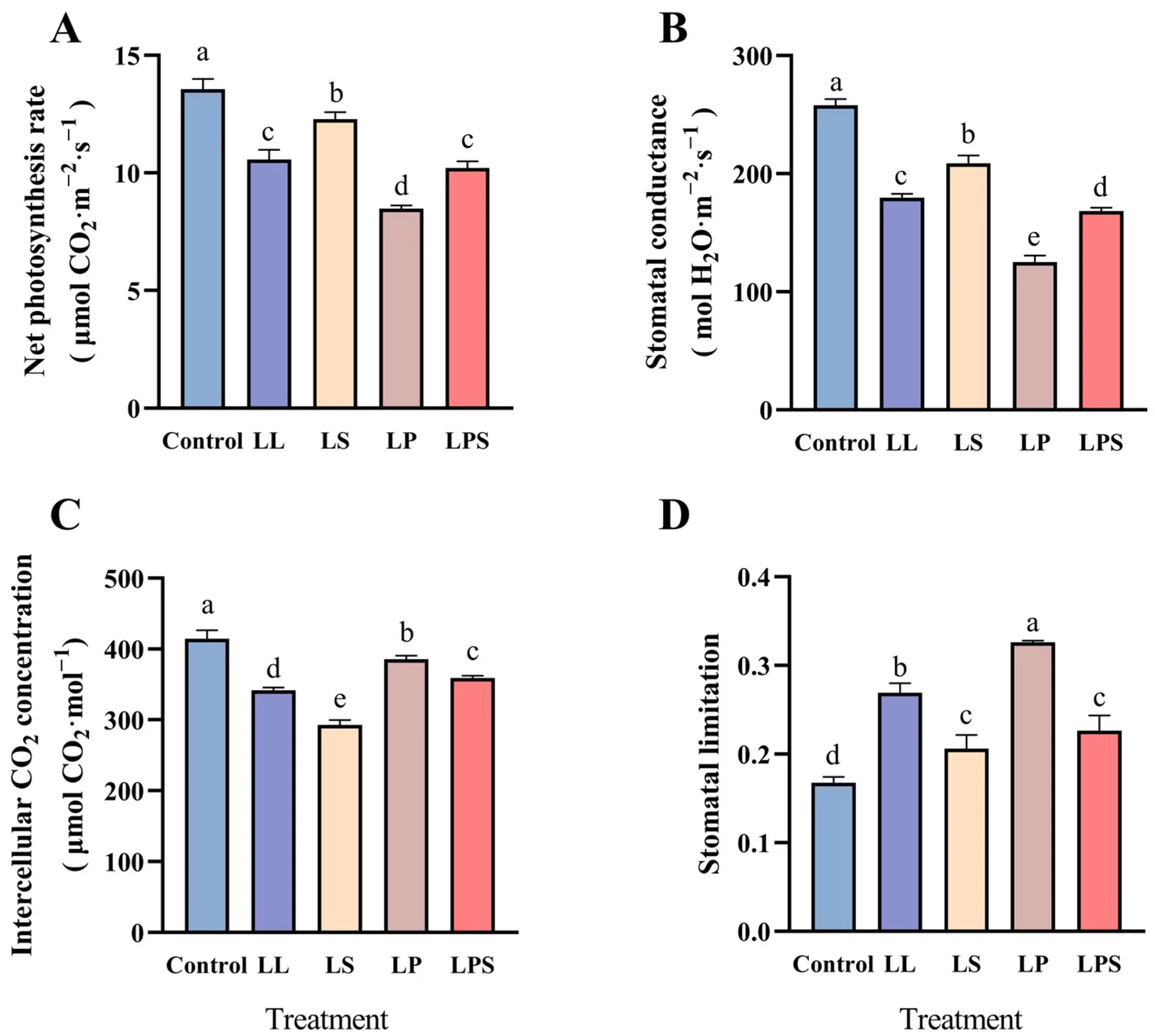
Effects of low light and nitric oxide on gas exchange parameters. Net photosynthetic rate (Pn, (**A**)), stomatal conductance (Gs, (**B**)), intercellular CO_2_ concentration (Ci, (**C**)), and stomatal limitation (Ls, (**D**)) were measured under control, low-light (LL), LL + SNP (LS), LL + PTIO (LP), and LL + PTIO + SNP (LPS) treatments. Data are mean ± SD (*n* = 3). Different letters indicate significant differences at *p* < 0.05 (Duncan’s test).

Photosynthetic parameters under different treatments are shown in Figure 3. Low-light treatment (LL) significantly reduced net photosynthetic rate (Pn), stomatal conductance (Gs), and intercellular CO_2_ concentration (Ci), while markedly increasing stomatal limitation (Ls) compared with the control. Relative to LL, exogenous application of SNP (LS) increased Pn and Gs by 16.1% each, while decreasing Ci and Ls by 14.5% and 23.4%, respectively. Notably, the decrease in Ci despite the increases in Pn and Gs suggests that the improvement in Pn was not attributable solely to stomatal regulation, but also involved alleviation of non-stomatal limitations. In contrast, treatment with PTIO (LP) further reduced Pn and Gs by 19.8% and 30.3% and increased Ci and Ls by 12.8% and 21.2%, respectively. The increase in Ci accompanied by a decrease in Pn under LP further suggests that non-stomatal limitations contributed substantially to the reduction in photosynthetic performance. Compared with LP, LPS significantly increased Pn and Gs while decreasing Ci and Ls, partially reversing the changes observed under LP.

### 2.4. Effects of Low Light and NO on PSI and PSII Photochemical Performance in Tomato Seedlings

**Figure 4:**
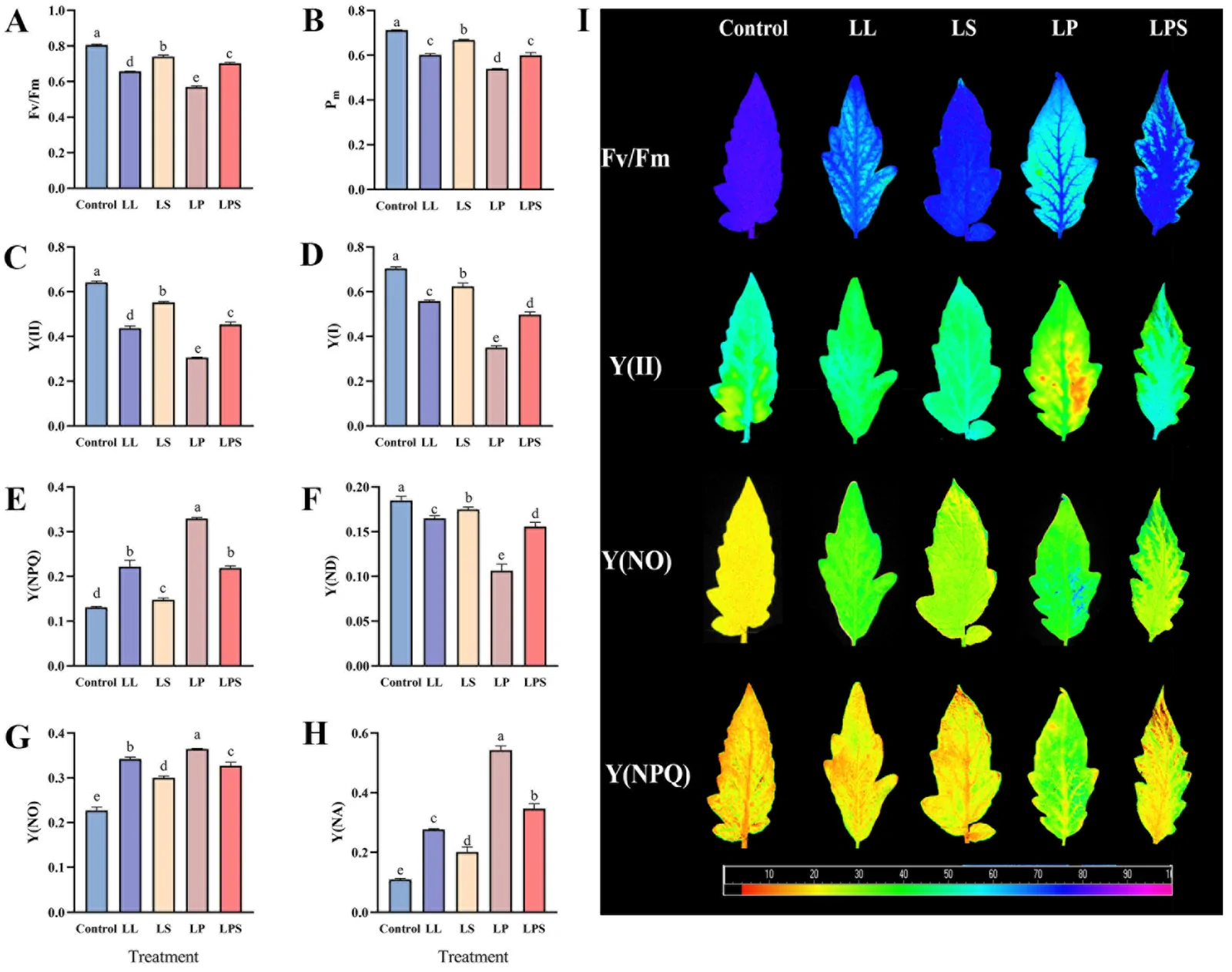
Effects of low light and nitric oxide on PSII and PSI photochemical performance. Maximum quantum efficiency of PSII (Fv/Fm, (**A**)), maximum P700 oxidation (Pm, (**B**)), effective quantum yield of PSII [Y(II), (**C**)], quantum yield of PSI photochemistry [Y(I), (**D**)], non-photochemical quenching [Y(NPQ), (**E**)], PSI donor-side limitation [Y(ND), (**F**)], non-regulated energy dissipation [Y(NO), (**G**)], PSI acceptor-side limitation [Y(NA), (**H**)], and representative false-color images of Fv/Fm, Y(II), Y(NO), and Y(NPQ) ((**I**)) were measured under control, low-light (LL), LL + SNP (LS), LL + PTIO (LP), and LL + PTIO + SNP (LPS) treatments. Data are mean ± SD (*n* = 3). Different letters indicate significant differences at *p* < 0.05 (Duncan’s test).

Chlorophyll fluorescence and P700 parameters reflecting the photochemical performance of PSII and PSI are presented in Figure 4. The maximum quantum efficiency of PSII (Fv/Fm), maximum P700 oxidation capacity (Pm), effective quantum yield of PSII [Y(II)], effective quantum yield of PSI [Y(I)], PSI donor-side limitation [Y(ND)], and PSI acceptor-side limitation [Y(NA)] were significantly affected by the low-light and NO treatments. Compared with the control, low light (LL) significantly decreased Fv/Fm, Pm, Y(II), Y(I), and Y(ND), while significantly increasing Y(NPQ), Y(NO), and Y(NA). Relative to LL, exogenous SNP application (LS) significantly increased Fv/Fm, Pm, Y(II), Y(I), and Y(ND), and decreased Y(NPQ), Y(NO), and Y(NA). In contrast, PTIO treatment (LP) significantly decreased Fv/Fm, Pm, Y(II), Y(I), and Y(ND) by 13.3%, 10.4%, 29.9%, 37.2%, and 35.6%, respectively, while increasing Y(NPQ), Y(NO), and Y(NA) by 48.6%, 6.7%, and 95.9%, respectively. Compared with LP, LPS treatment significantly increased Fv/Fm, Pm, Y(II), Y(I), and Y(ND) by 23.5%, 11.1%, 48.4%, 42.1%, and 46.6%, respectively, while decreasing Y(NPQ), Y(NO), and Y(NA) by 33.5%, 10.3%, and 36.2%, respectively.

**Figure 5:**
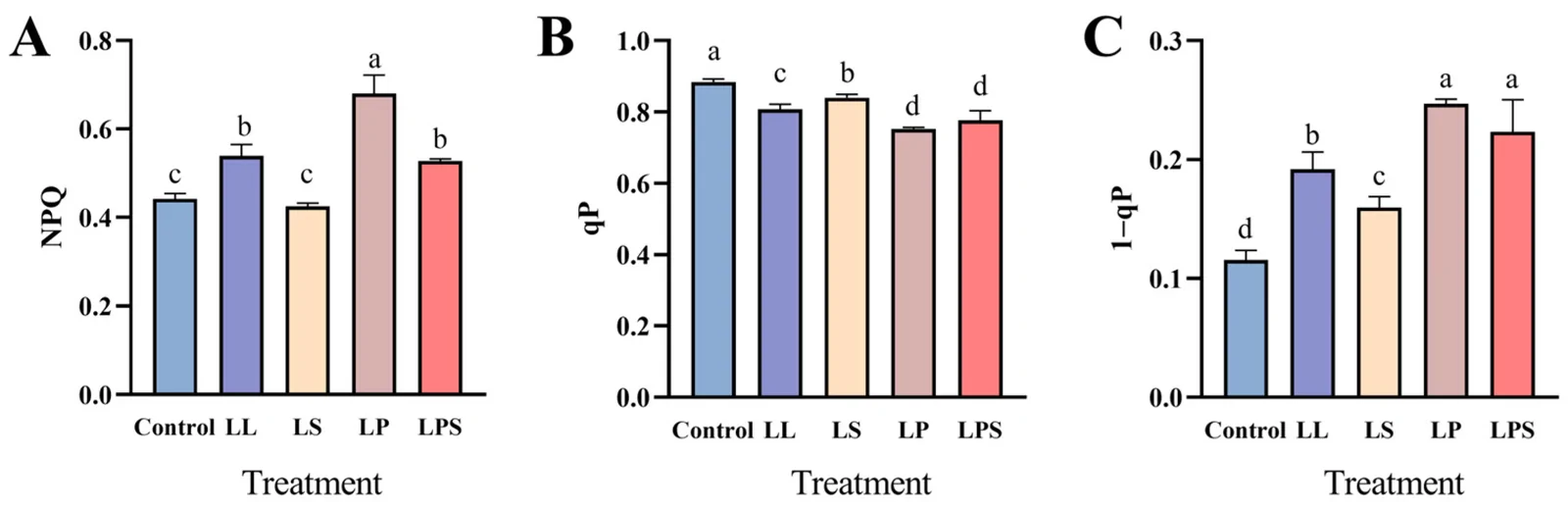
Effects of low light and nitric oxide on PSII energy partitioning. Non-photochemical quenching (NPQ, (**A**)), photochemical quenching (qP, (**B**)), and excitation pressure (1 − qP, (**C**)) were measured under control, low-light (LL), LL + SNP (LS), LL + PTIO (LP), and LL + PTIO + SNP (LPS) treatments. Data are mean ± SD (*n* = 3). Different letters indicate significant differences at *p* < 0.05 (Duncan’s test).

Changes in PSII energy partitioning parameters are shown in Figure 5. Compared with the control, LL significantly increased non-photochemical quenching (NPQ) and excitation pressure (1 − qP), while significantly decreasing photochemical quenching (qP). Relative to LL, SNP treatment (LS) significantly decreased NPQ (21.2%) and 1 − qP (16.8%) and increased qP (4.0%). Conversely, PTIO treatment (LP) significantly increased NPQ (26.2%) and 1 − qP (28.6%) and decreased qP (6.8%). Compared with LP, LPS treatment significantly reduced NPQ, whereas no significant differences were observed in qP or 1 − qP.

### 2.5. Absorbed Light Allocation Between PSI and PSII

**Figure 6:**
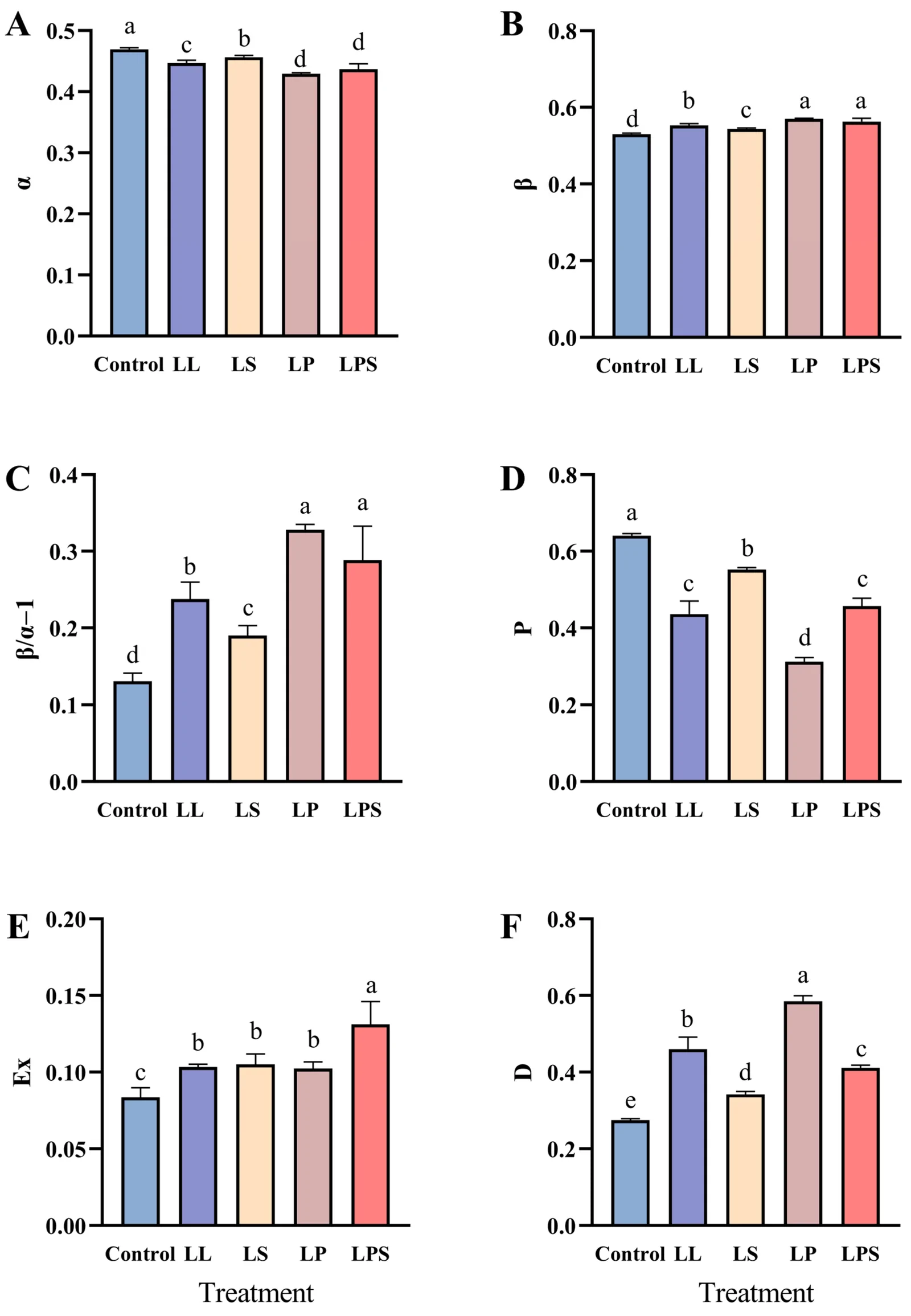
Effects of low light and nitric oxide on excitation energy distribution between PSI and PSII. Fraction of absorbed light energy in PSI (α, (**A**)) and PSII (β, (**B**)), β/α − 1 ratio (**C**), fraction of energy used in photochemistry (P, (**D**)), excess excitation energy (Ex, (**E**)), and thermal dissipation (**D**,**F**) were measured under control, low-light (LL), LL + SNP (LS), LL + PTIO (LP), and LL + PTIO + SNP (LPS) treatments. Data are mean ± SD (*n* = 3). Different letters indicate significant differences at *p* < 0.05 (Duncan’s test).

Changes in absorbed light energy distribution parameters are shown in Figure 6. The fraction of absorbed light energy distributed to PSI (α), fraction distributed to PSII (β), imbalance parameter between PSI and PSII (β/α − 1), photochemical energy utilization (P), excess excitation energy (Ex), and thermal dissipation (D) were significantly affected by low-light and NO treatments. Compared with the control, low-light treatment (LL) significantly decreased α and P, while significantly increasing β, β/α − 1, Ex, and D. Relative to LL, exogenous SNP (LS) application significantly increased α and P, while decreasing β, β/α − 1, and D, whereas no significant change was observed in Ex. In contrast, PTIO treatment (LP) markedly reduced α and P and significantly increased β, β/α − 1, and D, with no significant effect on Ex. Compared with LP, LPS treatment did not significantly affect α, β, or β/α − 1, but significantly increased P and Ex and decreased D.

### 2.6. Effects of Low Light and NO on Electron Transport and Cyclic Electron Flow

**Figure 7:**
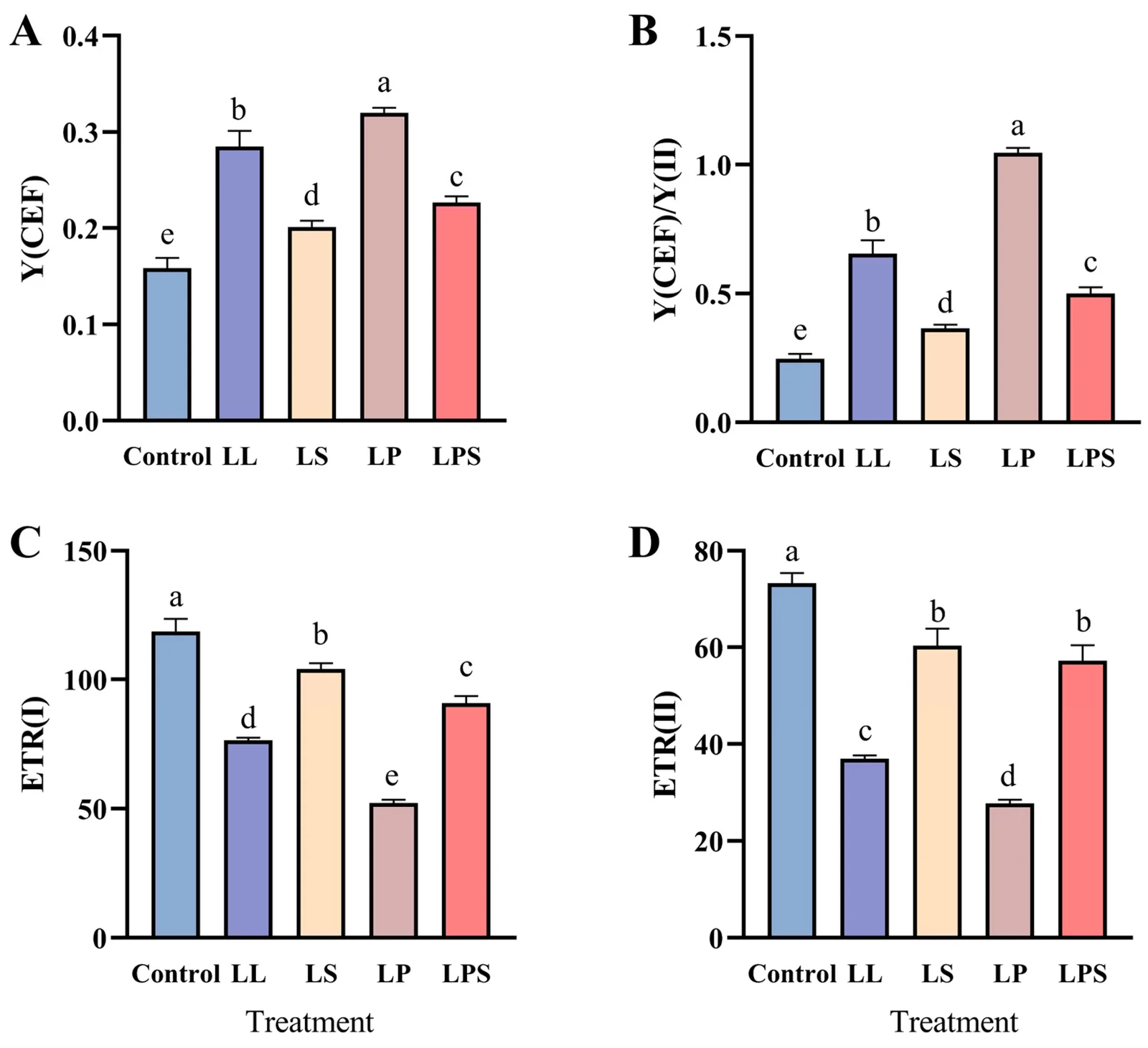
Effects of low light and nitric oxide on cyclic electron flow and electron transport rates. Quantum yield of cyclic electron flow [Y(CEF), (**A**)], proportion of CEF to PSII electron flow [Y(CEF)/Y(II), (**B**)], electron transport rate of PSI [ETR(I), (**C**)], and electron transport rate of PSII [ETR(II), (**D**)] were measured under control, low-light (LL), LL + SNP (LS), LL + PTIO (LP), and LL + PTIO + SNP (LPS) treatments. Data are mean ± SD (*n* = 3). Different letters indicate significant differences at *p* < 0.05 (Duncan’s test).

Changes in electron transport parameters are presented in Figure 7. The quantum yield of cyclic electron flow [Y(CEF)], the ratio of cyclic to linear electron flow [Y(CEF)/Y(II)], the electron transport rate of PSI [ETR(I)], and the electron transport rate of PSII [ETR(II)] were significantly influenced by low-light and NO treatments. Compared with the control, low-light treatment (LL) significantly increased Y(CEF) by 79.6% and Y(CEF)/Y(II) by 164.4%, while significantly decreasing ETR(I) and ETR(II) by 35.6% and 49.5%, respectively. Relative to LL, exogenous SNP application (LS) significantly decreased Y(CEF) and Y(CEF)/Y(II), while significantly increasing ETR(I) and ETR(II). In contrast, PTIO treatment (LP) further increased Y(CEF) and Y(CEF)/Y(II) under low-light conditions and significantly reduced ETR(I) and ETR(II). Compared with LP, LPS treatment significantly reversed these changes, decreasing Y(CEF) and Y(CEF)/Y(II) and increasing ETR(I) and ETR(II).

### 2.7. Effects of Low Light and NO on ROS Accumulation and Antioxidant Enzyme Activities in Tomato Seedlings

**Figure 8:**
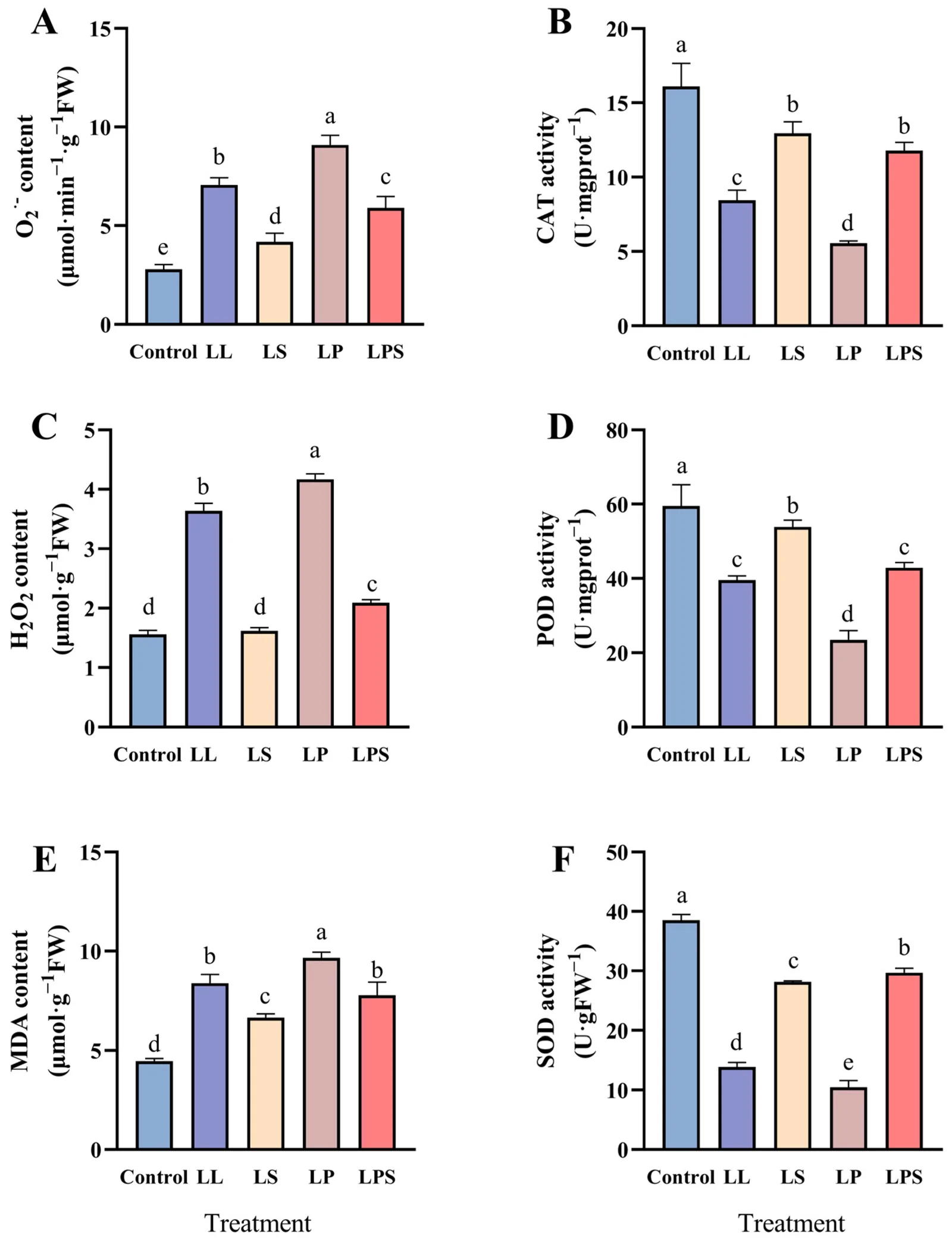
Effects of low light and nitric oxide on reactive oxygen species and antioxidant enzyme activities. Superoxide anion (O_2_·^−^, (**A**)), hydrogen peroxide (H_2_O_2_, (**C**)), and malondialdehyde (MDA, (**E**)) contents, together with catalase (CAT, (**B**)), peroxidase (POD, (**D**)), and superoxide dismutase (SOD, (**F**)) activities, were measured under control, low-light (LL), LL + SNP (LS), LL + PTIO (LP), and LL + PTIO + SNP (LPS) treatments. Data are mean ± SD (*n* = 3). Different letters indicate significant differences at *p* < 0.05 (Duncan’s test).

As shown in Figure 8, low-light treatment (LL) significantly increased the contents of superoxide anion (O_2_^·−^), hydrogen peroxide (H_2_O_2_), and malondialdehyde (MDA) compared with the control, while activities of catalase (CAT), peroxidase (POD), and superoxide dismutase (SOD) were markedly decreased. Relative to LL, exogenous application of SNP (LS) significantly reduced O_2_^·−^, H_2_O_2_, and MDA levels, and enhanced the activities of CAT, POD, and SOD. In contrast, treatment with the NO scavenger PTIO (LP) further elevated O_2_^·−^ by 28.7%, H_2_O_2_ by 15.6%, and MDA by 15.2%, while decreasing CAT, POD, and SOD activities by 34.2%, 40.6%, and 24.7%, respectively. Compared with LP, LPS treatment significantly mitigated these effects, reducing ROS accumulation and restoring antioxidant enzyme activities.

## 3. Discussion

Low light has become an important environmental constraint limiting crop productivity, particularly in regions characterized by frequent overcast and prolonged rainy conditions. Insufficient irradiance restricts photosynthetic carbon assimilation and ultimately suppresses plant growth and biomass accumulation. Plant growth performance is widely regarded as an integrated indicator of stress response [28]. Previous studies have shown that low light reduces biomass accumulation in cucumber [29] and tomato [30] and inhibits root and stem development in watermelon [31]. Consistent with these findings, low-light treatment markedly inhibited growth and reduced biomass accumulation in tomato seedlings in the present study (Figure 1). Nitric oxide (NO) is an important signaling molecule involved in plant adaptation to abiotic stresses, including low-light conditions [32]. In the present study, exogenous application of the NO donor sodium nitroprusside (SNP) significantly alleviated low-light-induced growth inhibition, whereas PTIO treatment further aggravated the reduction in biomass. Notably, subsequent application of SNP following PTIO treatment (LPS) partially restored growth under low-light conditions. Similar studies have shown that SNP can alleviate the adverse effects of low-light conditions on plant growth, while the effects of SNP under non-stressful light conditions may be relatively limited. For example, in pepper seedlings grown under normal illumination, SNP treatment did not significantly affect plant height, stem diameter, or leaf area compared with the untreated control, although some antioxidant-related responses were altered [26]. In addition, previous studies have reported that PTIO alone may have limited effects on several antioxidant parameters under non-stress conditions [33]. These findings suggest that the growth-promoting effects observed with SNP in the present study were more evident under low-light stress, although treatment-specific effects of SNP or PTIO under control irradiance cannot be completely excluded because corresponding SNP- and PTIO-treated control-light groups are not included in our experimental design. Therefore, the present results are interpreted primarily as the effects of SNP and PTIO treatments under low-light conditions rather than as direct evidence of changes in endogenous NO levels. It should also be noted that SNP may generate cyanide and iron-containing species during its decomposition in addition to releasing NO [34], and these additional products may contribute to the observed physiological responses. Photosynthesis is the primary source of energy for plant growth, and chlorophyll content directly determines light-harvesting efficiency. In this study, low light significantly reduced chlorophyll accumulation (Figure 2), which is consistent with previous reports that insufficient irradiance accelerates chlorophyll degradation or suppresses its biosynthesis [35,36]. NO application alleviated chlorophyll loss, whereas PTIO exacerbated pigment reduction, suggesting that NO contributes to maintaining chlorophyll stability under low-light conditions. Although low light often promotes LHCII accumulation as an acclimatory response in many land plants [37], α was reduced in LL plants in the present study. It should be noted that α represents the effective fraction of absorbed excitation energy allocated to PSI rather than the absolute abundance of LHCII [38,39]. Therefore, the reduction in α does not necessarily contradict an increase in antenna proteins but may instead indicate impaired excitation energy transfer and reduced photochemical utilization of absorbed light under prolonged low-light stress [11]. Reduced PSII photochemical utilization and increased excitation pressure may limit the effective use of absorbed energy despite potential increases in antenna abundance [16]. The partial recovery of α after NO application suggests that NO contributed to maintaining a more balanced excitation energy distribution between PSII and PSI. Beyond its effects on photosynthetic processes, NO can also regulate protein function through post-translational modifications [40], particularly S-nitrosation of cysteine residues, which may provide an additional molecular mechanism underlying NO-mediated stress responses [41]. Photosynthetic decline under low light is not solely attributable to pigment reduction but also involves both stomatal and non-stomatal limitations. Reduced Gs together with changes in Ci suggested that stomatal limitation contributed, at least in part, to the decline in Pn under low light (Figure 3). However, the recovery of Pn following NO application was not accompanied by proportional changes in Ci, indicating that improvements in CO_2_ diffusion alone could not fully explain the enhanced photosynthetic performance. Therefore, the beneficial effects of NO are likely attributable, at least in part, to non-stomatal processes associated with the maintenance of photosystem function and electron transport. Since non-stomatal limitations often arise from disturbances in photochemical processes and electron transport within both photosystems, photochemical parameters of PSII (chlorophyll fluorescence) and PSI (P700 redox kinetics) were further analyzed.

Low-light stress significantly decreased Fv/Fm and Pm (Figure 4), indicating suppression of both PSII and PSI activities. The decline in Y(II) and qP, together with increased 1 − qP and Y(NO) (Figure 5), suggests enhanced excitation pressure on PSII reaction centers and impaired electron transport. Elevated Y(NO) further indicates increased non-regulated energy dissipation, which may reflect destabilization of PSII supercomplexes or interference with D1 protein turnover [42,43]. Exogenous NO partially restored Y(II) and qP while reducing Y(NO), whereas PTIO weakened these protective effects. Increased NPQ and reduced excitation pressure after NO treatment suggest that NO enhances regulated thermal dissipation and alleviates photoinhibitory stress on PSII. Previous studies have demonstrated that NO participates in the xanthophyll cycle and promotes D1 protein turnover under stress [44,45], supporting the protective role observed here. Compared with PSII, PSI photoinhibition is often more detrimental due to its slower recovery and potential to induce secondary oxidative damage [46]. Under low light, reduced Y(I) and Y(ND) accompanied by increased Y(NA) indicate acceptor-side limitation and over-reduction of P700, suggesting impaired PSI activity. NO treatment partially reversed these changes, implying that NO helps maintain P700 in a relatively oxidized state and reduces PSI photodamage. Balanced excitation energy distribution between PSI and PSII is essential for efficient linear electron flow. State transition serves as a rapid acclimation mechanism to adjust energy allocation [46]. Decreases in α and P, together with increases in β, Ex, and β/α − 1 under low light (Figure 6), indicate disrupted energy distribution and increased excitation pressure on PSII. PTIO aggravated this imbalance, whereas NO improved qP and redirected energy toward photochemical utilization, thereby enhancing photosystem coordination.

Cyclic electron flow (CEF) around PSI is an important alternative electron transport pathway involved in maintaining photosynthetic electron balance under stress conditions [47]. In the present study, low light significantly increased Y(CEF) and Y(CEF)/Y(II), whereas ETR(I) and ETR(II) were markedly decreased (Figure 7). This pattern suggests an adjustment in electron partitioning under low-light conditions accompanied by reduced linear electron transport. Notably, SNP treatment decreased Y(CEF) and Y(CEF)/Y(II) but increased ETR(I) and ETR(II), whereas PTIO produced opposite responses. The LPS treatment largely reversed the effects observed in the PTIO treatment. These results suggest that the increased relative CEF contribution under low light, particularly in the PTIO treatment, may reflect an adjustment in electron partitioning associated with impaired linear electron transport, rather than providing direct evidence of enhanced CEF activity.

Disruption of photosynthetic electron transport can disturb cellular redox homeostasis and promote the accumulation of reactive oxygen species (ROS), including superoxide anion (O_2_·^−^) and hydrogen peroxide (H_2_O_2_) [48]. The balance between excitation energy utilization, electron transport, ROS production, and antioxidant defense is closely associated with the stability and repair of photosynthetic components under stress conditions [49]. Excessive ROS accumulation can cause oxidative damage to cellular lipids, proteins, and other macromolecules, thereby impairing cellular and photosynthetic functions [50]. Malondialdehyde (MDA), a product of membrane lipid peroxidation, is therefore widely used as a biochemical indicator of oxidative membrane damage [51]. Plants rely on coordinated antioxidant defense systems to control ROS accumulation. Superoxide dismutase (SOD) catalyzes the dismutation of O_2_·^−^ to H_2_O_2_, whereas catalase (CAT) and peroxidases (POD/POX) contribute to the subsequent detoxification of H_2_O_2_. Therefore, measurements of O_2_·^−^ and H_2_O_2_ contents together with MDA accumulation and the activities of SOD, POD, and CAT provide complementary information on ROS status, oxidative damage, and antioxidant defense capacity [52]. NO is also involved in the regulation of redox homeostasis and antioxidant responses under abiotic stress. Low light increased ROS accumulation and suppressed antioxidant enzyme activities (Figure 8), indicating an imbalance between ROS production and scavenging. In contrast, SNP treatment enhanced antioxidant capacity and reduced oxidative damage. Together with the recovery of ETR(I) and ETR(II), these results suggest that the protective effect of SNP under low light was mainly associated with improved photosystem function, restoration of linear electron transport, and maintenance of redox homeostasis, rather than stimulation of CEF.

## 4. Materials and Methods

### 4.1. Plant Material and Experimental Design

The tomato (*Solanum lycopersicum* L.) cultivar ‘Zhongshu No. 4’ was used as the experimental material. Seeds were germinated and sown in 50-cell plug trays, and seedlings were cultivated in a greenhouse at Kaili University, Guizhou Province, China. When the seedlings reached the two-leaf stage, they were transplanted into plastic pots (13 cm × 13 cm) filled with a standard growth substrate. During cultivation, water and fertilizer were applied uniformly. The average day/night temperatures in the greenhouse were maintained at 25 °C/15 °C with a relative humidity of approximately 60%. When the plants reached the four-leaf stage, the low-light treatments were initiated. The low-light environment was established in a controlled growth chamber using high-pressure sodium lamps as the light source. Prior to treatment, all seedlings were transferred to the growth chamber and acclimated for five days under control-light conditions. Five treatments were applied: control (450 ± 30 μmol m^−2^ s^−1^, foliar spray with distilled water), LL (80 ± 10 μmol m^−2^ s^−1^, foliar spray with distilled water), LS (80 ± 10 μmol m^−2^ s^−1^, foliar spray with 100 μmol L^−1^ sodium nitroprusside, SNP), LP (80 ± 10 μmol m^−2^ s^−1^, foliar spray with 50 μmol L^−1^ PTIO), and LPS (80 ± 10 μmol m^−2^ s^−1^, foliar application of 50 μmol L^−1^ PTIO followed by foliar application of 100 μmol L^−1^ SNP). SNP was used as the nitric oxide (NO) donor, and PTIO (2-phenyl-4,4,5,5-tetramethylimidazoline-1-oxyl-3-oxide) served as a specific NO scavenger.

The control irradiance (450 ± 30 μmol m^−2^ s^−1^) was selected to represent the moderate-light conditions commonly used for tomato seedling cultivation in controlled-environment studies, whereas the low-light treatment (80 ± 10 μmol m^−2^ s^−1^) was adopted to simulate prolonged cloudy conditions frequently encountered during tomato production in protected cultivation. These irradiance levels are comparable to those used in previous studies on tomato seedlings, in which control plants were grown under 450–500 μmol m^−2^ s^−1^ and low-light treatments were imposed at approximately 75–100 μmol m^−2^ s^−1^ to investigate photosynthetic acclimation and stress responses [12,53].

All solutions were applied by foliar spraying once daily throughout the treatment period. The experiment was arranged in a randomized block design with three independent biological replicates per treatment (*n* = 3). Each biological replicate consisted of five plants, and the biological replicate was considered the experimental unit for statistical analysis. Leaf samples were collected on the ninth day after the initiation of treatments for subsequent physiological and biochemical analyses.

### 4.2. Determination of Plant Growth Parameters

Plant growth parameters including plant height, stem diameter, shoot fresh weight, root fresh weight, shoot dry weight, and root dry weight were determined after nine days of treatment. Plant height was measured as the distance from the cotyledonary node to the shoot apical meristem, while stem diameter was measured at the cotyledonary node using a digital caliper. For biomass determination, whole plants were carefully uprooted and gently washed with tap water to remove surface substrate particles, followed by rinsing with deionized water. After removing excess surface moisture with absorbent paper, shoots and roots were separated and their fresh weights were recorded. The samples were then oven-dried at 105 °C for 15 min to terminate metabolic activity, followed by drying at 75 °C until a constant weight was achieved, and the dry weights were subsequently measured [54].

### 4.3. Determination of Chlorophyll Content

Chlorophyll content was determined according to the method described by Şaşkın [55]. Briefly, 0.5 g of fresh leaf tissue was sampled from each treatment and homogenized thoroughly in 5 mL of 80% (*v*/*v*) acetone. The homogenate was filtered through qualitative filter paper, and the filtrate was collected in light-proof tubes to prevent pigment degradation. The absorbance of the extract was measured using a spectrophotometer at 663.5 nm for chlorophyll a (*Chl a*) and 645 nm for chlorophyll b (*Chl b*), with 80% acetone used as the blank control. Chlorophyll concentrations were calculated using the corresponding equations described by Şaşkın [55]. Total chlorophyll content (*Chl a* + *b*) was calculated as the sum of *Chl a* and *Chl b*, and the chlorophyll a/b ratio (*Chl a*/*b*) was calculated as *Chl a* divided by *Chl b*. The results were first expressed as mg L^−1^ and subsequently converted to mg·g^−1^ fresh weight (FW).

### 4.4. Determination of Photosynthetic Parameters

Gas exchange parameters, including net photosynthetic rate (Pn), stomatal conductance (Gs), and intercellular CO_2_ concentration (Ci), were measured using a portable photosynthesis system (LI-6400, LI-COR Inc., Lincoln, NE, USA). Measurements were conducted on the fully expanded third leaves from the top between 09:00 and 11:00 h under controlled conditions with a photosynthetic photon flux density (PPFD) of 800 μmol m^−2^ s^−1^ provided by a red–blue LED light source, leaf chamber CO_2_ concentration of 400 μmol mol^−1^, leaf temperature of 25 ± 1 °C, relative humidity of 60–70%, and flow rate of 500 μmol s^−1^. The stomatal limitation value (Ls) was calculated according to the method described by Chen [56]. The gas exchange measurements were performed at 800 μmol m^−2^ s^−1^ PPFD to assess the photosynthetic capacity of leaves following the respective light treatments rather than their steady-state photosynthetic performance under growth irradiance. Similar measurement conditions have been widely used to assess photosynthetic acclimation to low-light stress [27,57].

### 4.5. Chlorophyll Fluorescence and P700 Measurements

Chlorophyll fluorescence parameters were determined using a pulse-amplitude modulated chlorophyll fluorescence imaging system (Imaging-PAM, Heinz Walz GmbH, Effeltrich, Germany) according to the protocol described by Baker [58]. Leaves were dark-adapted for 30 min before measurements. After dark adaptation, chlorophyll fluorescence and P700 parameters were measured under a fixed actinic light intensity of 280 μmol photons m^−2^ s^−1^, which was consistently applied to all treatments. All measurements were performed using identical measuring settings for all treatments to ensure comparability among samples. The maximum quantum efficiency of PSII (Fv/Fm), effective PSII quantum yield [Y(II)], photochemical quenching (qP), non-photochemical quenching (NPQ), and the quantum yields of regulated [Y(NPQ)] and non-regulated [Y(NO)] energy dissipation were obtained from the Imaging-PAM software (ImagingWin, v2.60, Walz, Effeltrich, Germany) based on the recorded fluorescence signals (Fo, Fm, Fs, Fm′, and Fo′). PSI redox parameters were determined simultaneously with a Dual-PAM-100 (Heinz Walz GmbH, Effeltrich, Germany) at room temperature (approximately 25 °C). The saturation pulse intensity and duration were set to 10,000 μmol photons m^−2^ s^−1^ and 300 ms, respectively. All electron transport rate (ETR) calculations for PSI and PSII were performed using the built-in software of the Dual-PAM-100 with fixed calculation parameters. The leaf absorptivity was set to 0.84, and the distribution ratio of absorbed light energy between PSI and PSII was set to 0.5. The effective quantum yield of PSI photochemistry [Y(I)], the donor-side limitation [Y(ND)], the acceptor-side limitation [Y(NA)], as well as the electron transport rates of PSI [ETR(I)] and PSII [ETR(II)] were automatically calculated by the Dual-PAM software based on P700 absorbance and chlorophyll fluorescence signals. The excitation energy distribution parameters between PSI and PSII, including the fraction of absorbed light energy utilized for photochemistry (P), excess excitation energy (Ex), thermal dissipation (D), and the deviation coefficient of excitation energy imbalance (β/α − 1), were subsequently calculated from Fv′/Fm′ and qP according to Demmig-Adams [59]. False-color images of Fv/Fm, Y(II), Y(NPQ), and Y(NO) were generated using the Imaging-PAM imaging software. The fluorescence images were displayed according to a color scale ranging from 0.000 (black) to 1.000 (purple), representing the spatial distribution of photosynthetic performance across the leaf surface.

### 4.6. Estimation of Cyclic Electron Flow (CEF)

Cyclic electron flow around PSI (CEF) was estimated based on the difference between the effective quantum yields of PSI and PSII, following the method described by Lu [57]. The relative CEF activity was calculated as:

Y(CEF) = Y(I) − Y(II)

The proportion of CEF to total PSII electron flow was expressed as:

Y(CEF)/Y(II) = [Y(I) − Y(II)]/Y(II)

where Y(I) and Y(II) represent the effective quantum yields of PSI and PSII, respectively, obtained from Dual-PAM measurements. These parameters were used to evaluate the contribution of cyclic electron transport to photosynthetic electron partitioning under different treatments.

### 4.7. Determination of Reactive Oxygen Species (ROS) and Antioxidant Enzyme Activities

Malondialdehyde (MDA) content was determined using the thiobarbituric acid (TBA) reaction, as described by Wang [60]. Hydrogen peroxide (H_2_O_2_) content was quantified spectrophotometrically according to the method of Velikova [61], while the superoxide anion (O_2_·^−^) production rate was determined following Zhang [62]. For antioxidant enzyme assays, fresh leaf tissues (0.5 g) were homogenized in ice-cold 50 mmol L^−1^ phosphate buffer (pH 7.8) and centrifuged at 12,000× *g* for 20 min at 4 °C. The resulting supernatant was used for the determination of superoxide dismutase (SOD), peroxidase (POD), and catalase (CAT) activities according to Rahmani [63].

### 4.8. Statistical Analysis

All experiments were performed with at least three independent biological replicates. Data were organized using Microsoft Excel and expressed as the mean ± standard deviation (SD). Statistical analyses were conducted using SPSS software (IBM SPSS Statistics, version 19.0, IBM Corp., Armonk, NY, USA). Differences among treatments were evaluated by one-way analysis of variance (ANOVA) followed by Duncan’s multiple range test. Differences were considered statistically significant at *p* < 0.05. Graphs were generated using GraphPad Prism (version 8.0, GraphPad Software, San Diego, CA, USA).

## 5. Conclusions

**Figure 9:**
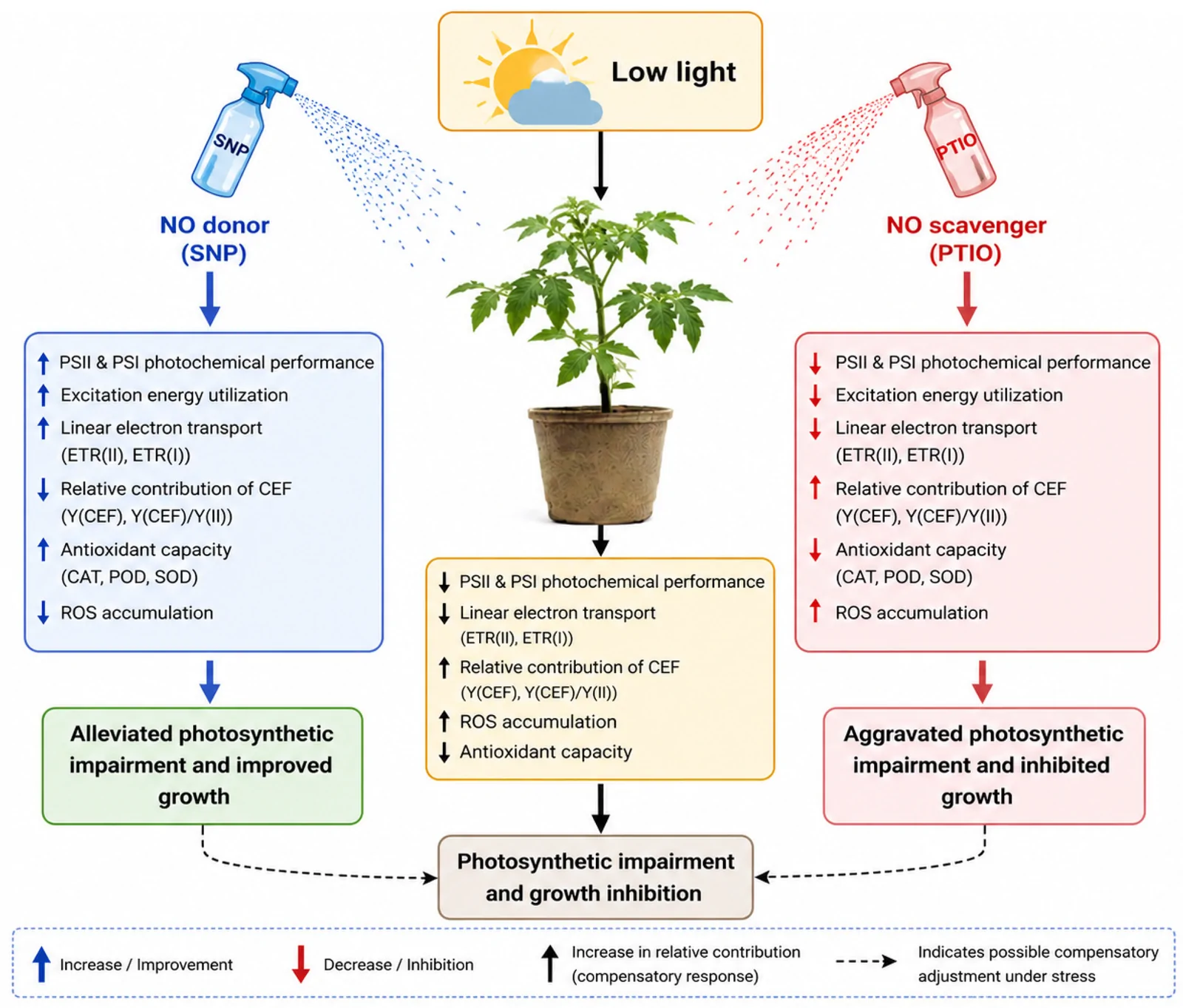
Schematic model of nitric oxide-mediated photoprotection under low-light stress in tomato seedlings.

In summary, prolonged low light impaired photosynthetic performance and PSI–PSII function, resulting in reduced electron transport, oxidative stress, and growth inhibition in tomato seedlings. Exogenous NO alleviated these effects by improving excitation energy utilization, maintaining PSI–PSII coordination, restoring linear electron transport, and enhancing antioxidant defenses (Figure 9). The increased relative CEF under low light, particularly following PTIO treatment, may reflect an adjustment in electron partitioning associated with impaired linear electron transport rather than a direct enhancement of CEF by NO. Overall, our findings indicate that NO contributes to the maintenance of photosynthetic and redox homeostasis under prolonged low-light conditions.

## Data Availability

The raw data supporting the conclusions of this article will be made available by the corresponding author upon reasonable request.
